# Obesity and non-alcoholic steatohepatitis in immunotherapy for hepatocellular carcinoma

**DOI:** 10.1007/s12072-023-10533-w

**Published:** 2023-05-15

**Authors:** Shuichiro Shiina, Hitoshi Maruyama, Maki Tobari, Tatsuya Yamashita

**Affiliations:** 1 Department of Gastroenterology, Juntendo University, 2-1-1, Hongo, Bunkyo-Ku, Tokyo, 113-8421 Japan; 2 Department of Gastroenterology, Kanazawa University, 13-1 Takara-Machi, Kanazawa, Ishikawa Japan

The atezolizumab plus bevacizumab combination therapy (Atezo/Beva) opened the door to immunotherapy for unresectable hepatocellular carcinoma (HCC) [1]. Following Atezo/Beva, three other combination immunotherapies, sintilimab plus a bevacizumab biosimilar (ORIENT-32) [2], tremelimumab plus durvalumab (HIMALAYA) [3], atezolizumab plus cabozantinib (COSMIC-312) [4], have shown survival benefits as first line systemic therapy for unresectable HCC. Systemic therapy for HCC is shifting toward immunotherapy.

A subgroup analysis in the IMbrave 150 study showed that Atezo/Beva demonstrated an overall survival (OS) benefit over sorafenib in patients with hepatitis B or hepatitis C, but not in patients with non-viral etiology [1, 5]. Thus, it has been argued that Atezo/Beva may not have a survival benefit in non-viral HCC. However, differences in clinical characteristics in the three groups were not explained. Furthermore, in non-alcoholic steatohepatitis (NASH)-related HCC, the aberrant T cell activation causing tissue damage results in impaired immune surveillance, which causes less response to immunotherapy based on basic research [6]. These findings suggest that immunotherapy has a limited effect for non-viral or NASH-related HCC. Several retrospective analyses have reported that Atezo/Beva may be less effective compared with lenvatinib in NASH–HCC [7, 8].

Vithayathil et al. [9] reported the effect of body mass index (BMI) in patients with HCC treated with Atezo/Bev. They analyzed 191 consecutive patients with unresectable HCC treated with Atezo/Beva from eight centers in seven countries. The cohort included 23.0% Child–Pugh class B. The progression-free survival (PFS) in this cohort was comparable to the result in IMbrave150 (median 6.7 months vs. 6.8 months). However, OS was relatively shorter than that in IMbrave 150 (14.9 months vs. 19.2 months) [1, 5, 9]. They divided the cohort by BMI into those with a BMI 25 or greater (overweight) and those with a BMI less than 25 (non-overweight). This is the definition of the World Health Organization classification of overweight and close to the median BMI in their cohort: the number of patients in each group was similar (97 non-overweight vs. 94 overweight). Regarding the baseline characteristics, although many baseline characteristics were similar between the two groups, the percentage of non-alcoholic fatty liver disease (NAFLD) was significantly higher in the overweight group than in the non-overweight group (19.2 vs. 7.2%). There were also significant differences in the rates of hepatitis B virus-positivity, macrovascular invasion, extrahepatic spread, and previous resection between the groups. In terms of efficacy, the overweight group had similar OS, PFS, overall response rate (ORR), and disease control rate (DCR) compared to the non-overweight group. The effects of the difference in the baseline characteristics on OS and PFS were analyzed by multivariate analysis. These statistical analyses concluded that overweight had no effect on OS and PFS, while liver function Child–Pugh class A or B had an effect on OS and PFS. Supplementary data showed their evaluation of different BMI classes (underweight, normal, overweight, and obese) on OS and PFS, but these BMI classes did not affect OS and PFS. Based on these results, it was concluded that BMI has no impact on the efficacy of Atezo/Beva in patients with unresectable HCC [9]. They also concluded that Atezo/Beva is effective in overweight HCC patients, including those with underlying NAFLD. The authors clearly showed comparable efficacy in both overweight and non-overweight groups. However, the conclusion may be an overestimate, since the percentage of NAFLD in the overweight group was only 19.2%. Most of the overweight group were patients without NAFLD. Moreover, the proportion of NASH remains unknown. While the results of this article are compelling, no conclusions can be drawn from these results regarding the effect of Atezo/Bev on NASH HCC.

There are interesting reports on the relationship between immunotherapy and obesity in cancer. Obesity is reaching pandemic proportions [10]. In general, excess body weight is associated with poor outcomes in cancer treatments. Obesity is associated with chronic low-grade inflammation, resulting in changes of the immune conditions of multiple organ systems. On the basis of the association between chronic inflammations and cancer, it is likely that obesity is correlated with increased risk and worse prognosis for various types of malignancies. Paradoxically, in most retrospective studies of melanoma [11], non-small cell lung cancer [12], and renal cell carcinoma, higher BMI has been associated with improved outcomes in patients treated with immunotherapies using immune checkpoints inhibitors (ICI), in what has been called the “obesity paradox.” The “obesity paradox” is the concept that immunotherapy, especially ICI-based immunotherapy, improves survival in obese cancer patients. The exact mechanism of the “obesity paradox” “remains to be elucidated. Elevated leptin levels may be one explanation. Obesity induces a high level of leptin production, which positively affects the function, metabolism, and survival of CD8^+^ tumor-infiltrating lymphocytes, induces the high expression of PD-1 on immune cells, and increases PD-1/PD-L1 interaction. Therefore, in obesity, anti-PD-1/PD-L1 therapy can block the increased PD-1/PD-L1 interaction and activate an immune response more effectively [13]. Although there are no data reporting this obesity paradox in immunotherapy of HCC, the overweight group in the study may accept a positive effect on the efficacy of immunotherapy [14]. Since adipokines such as leptin, adiponectin, and lecithin are said to be involved in obesity-related HCC [15], including NASH–HCC, these adipokines may also affect the microenvironment of HCC. They may play some roles in the effectiveness of immunotherapy.

The issue of NASH–HCC and immunotherapy, which is currently the focus of much attention, can only be analyzed in cohorts divided by BMI or by the presence or absence of obesity, although many confounding factors may be involved. Obesity includes NAFLD and non-NAFLD, and NAFLD includes NASH and non-NASH. In the context of obesity, the “obesity paradox” condition may be involved [16]. In the context of NASH–HCC, the progression of NASH is associated with decreased hepatic reserve, which has a strong prognostic impact. The most direct solution would be to compare Atezo/Beva with other therapies such as lenvatinib or tremelimumab plus durvalumab in a randomized controlled trial in histologically diagnosed NASH-–HCC only. However, it would be difficult to achieve in practice. Since multiple immunotherapies are currently being introduced, crossover between immunotherapies is likely to occur. The issue of immunotherapy with obesity and NASH–HCC will continue to be debated from various angles.

## Data Availability

Data will be available in the future but not at present.
